# Electroacupuncture stimulation improves pulmonary fibrosis by modulating ferroptosis in rats: multiscale analysis of transcriptome and proteome

**DOI:** 10.1186/s13020-025-01184-0

**Published:** 2025-08-20

**Authors:** Yange Tian, Qinghua Song, Ruilong Lu, Yan Du, Zhiguang Qiu, Yixi Liao, Bo Wang, Jiansheng Li

**Affiliations:** 1 Henan University of Chinese Medicine, 156 Jinshui East Road, Jinshui District, Zhengzhou, 450046 Henan Province China; 2 Collaborative Innovation Center for Chinese Medicine and Respiratory Diseases Co-Constructed By Henan Province & Education Ministry of P.R. China, Zhengzhou, 450046 China; 3 The First Affiliated Hospital of Henan University of Chinese Medicine, Zhengzhou, 450046 China

**Keywords:** Pulmonary fibrosis, Electroacupuncture, Proteomic, Transcriptomic, Ferroptosis

## Abstract

**Background:**

Idiopathic Pulmonary fibrosis (IPF) is a progressive lung disease with poor survival. Electroacupuncture has been proven to improve dyspnea in IPF patients, but the mechanism remains unclear.

**Methods:**

The IPF rat model was established by intratracheal instillation of bleomycin. Electroacupuncture was performed 3 times a week for 4 weeks. Lung function and lung histopathology were tested to evaluate the respiratory movements and lung damage. Collagen I (COL-I), α-smooth muscle actin (α-SMA) and hydroxyproline (HYP) were measured to evaluate fiber deposition. Characterization of gene and protein expression profiles in IPF rat was recognized by integrated proteomic and transcriptomic. WGCNA and GSEA were used to identify the key modules and signaling pathways of electroacupuncture against IPF.

**Results:**

Electroacupuncture improved vital capacity, RI, Cdyn, the alveolar rupture and fibrous tissue deposition, and reduced the expression of α-SMA, COL-I, and HYP. 1104 differentially expressed genes and 391 proteins were identified which were reversed by electroacupuncture. Two modules were obtained and functional analysis showed ferroptosis, PI3K-AKT and FoxO signaling pathway were significantly enriched. Genes and proteins with strong correlations were screened out, and functional analysis showed ferroptosis and glutathione metabolism were significantly enriched. Electroacupuncture reduced the levels of Fe^3+^, Fe^2+^, LPO and MDA in the lung tissue of PF rats and increased the levels of GSH and SOD. Further, electroacupuncture improved the mitochondrial swelling of alveolar epithelial cells in PF rats. Also, electroacupuncture inhibited the level of p-Akt and p-FoxO3.

**Conclusions:**

Electroacupuncture regulated ferroptosis to improve IPF via inhibiting PI3K-AKT and FoxO signaling.

## Introduction

Idiopathic pulmonary fibrosis (IPF) is a prototype of chronic, progressive, and fibrotic lung disease [1]. The mechanisms of fibrosis in IPF remain elusive and are often thought to be related to genetically linked recurrent microdamage of the alveolar epithelium, followed by an abnormal repair response characterized by excessive collagen deposition [2]. The physical, social and psychological burden of IPF is huge [3], and the survival period of untreated IPF is only 3–5 years [4]. Pirfenidone and nintedanib are approved as therapeutic drugs for IPF because of their beneficial effect in slowing down the decline of lung function [5]. However, they cannot cure IPF and some patients cannot tolerate them for long-term use [6, 7]. Therefore, it is particularly important to explore new therapeutic strategies against IPF.

Electroacupuncture, as a characteristic treatment method of traditional Chinese medicine, has been proven to be able to regulate body functions and restore homeostasis by stimulating body specific areas (acupoints) [8]. Increasing evidence shows that electroacupuncture can treat various diseases by stimulating the nervous system [9], gastrointestinal motility [10] and endocrinium [11]. Matsumoto-Miyazaki J et al. demonstrated the beneficial effect of acupuncture in treating IPF patients. After 10 weeks of acupuncture treatment, the blood oxygen saturation of IPF patient increased, and the symptoms of dyspnea and 6-min walking distance were significantly improved [12]. Xie et al. integrated research on acupuncture treatment of IPF in 8 databases and found that acupuncture could improve dyspnea, limited movement, and damage to health conditions in IPF patients, providing evidence for the effectiveness and safety of acupuncture [13]. Similarly, our previous research found that electroacupuncture could effectively improve the reduced lung function and lung structural damage in rats caused by bleomycin (BLM), but the mechanism was unclear.

In recent years, transcriptomics has been widely used to identify key genes or important biological processes in diseases [14]. Increasing evidence demonstrates the efficiency and accuracy of transcriptomics in screening hub genes [15]. By analyzing the transcriptome of lung tissue of 116 IPF patients, Brownstein AJ et al. identifies key genes associated with pulmonary hypertension in IPF patients, and reveals the potential mechanism of IPF with pulmonary hypertension [16]. Benefit from the rapid development of mass spectrometry technology, proteomics has become a powerful tool capable of identification of biomarkers [17], characterization of extracellular vesicles [18] and prediction of disease prognosis [19]. The main purpose of transcriptomics is to study the expression changes of related genes, but it cannot well reflect the biological processes of cells, while proteomics can better explain the biogenesis of cells [20]. By integrating the lung tissue transcriptome and plasma proteome of IPF patients, Pitchumani et al. found that IPF patient samples exhibited strong chemotaxis, enrichment of tumor infiltration and mast cell migration pathways, and downregulation of extracellular matrix (ECM) degradation [21].

In this study, we aimed to identify BLM-induced differential expression profiles in rat lung tissue through transcriptomics and proteomics. Then, we explored the key genes and important biological processes of electroacupuncture in treating pulmonary fibrosis through multi-omics methods to explain the potential mechanism of electroacupuncture.

## Materials and methods

### Reagents and chemicals

BLM hydrochloride for injection (S1214) was provided by Japan Co., Ltd. Isoflurane (R510-22-10) was purchased from Shenzhen Rayward Life Technology Co., LTD. Acupuncture needles (0.30 × 13 mm) were purchased from Suzhou Acupuncture Supplies Co., Ltd. Cole hematoxylin staining solution (G1140), eosin staining solution (G1140) and Masson staining solution (G1340) were purchased from Beijing Solarbio Biotechnology Co., Ltd. Hydroxyproline (HYP) kit (BC0255) was purchased from Nanjing Jiancheng Technology Co., Ltd. Rat interleukin (IL)−4 ELISA kit (E-EL-R0014c) and tumor necrosis factor (TNF)-α ELISA kit (E-EL-R2856c) were purchased from Wuhan Elabscience Biotechnology Co., Ltd. Collagen (COL)-I antibody (AF7001) was purchased from Affinity Company in Australia. α- smooth muscle actin (SMA) antibody (19245 T) was purchased from Germany CST Company. The protease inhibitor cocktail and BCA protein quantification kit were purchased from Thermo Fisher Scientific Inc. (Waltham, MA, USA). Sequencing-grade modified trypsin was purchased from Promega Co. (Madison, WI, USA). C18 cartridge columns were purchased from Anpel Laboratory Technologies Co., Ltd. (Shanghai, China). HPLC-grade acetonitrile was purchased from Merck (Darmstadt, Germany).

### Establishment of pulmonary fibrosis rat model

24 SPF SD male rats were purchased from Sibeifu Biotechnology Co., LTD (Beijing, China). During the entire experiment, rats were kept at 25 ± 2 °C and a 12-h light/dark cycle, with free access to food and water. The rats were randomly divided into normal group, model group and electroacupuncture group with 8 in each group. After 1 week of adaptive feeding, a rat model of pulmonary fibrosis was established by a single intratracheal instillation of BLM [22]. The successful establishment of the PF rat model was determined by assessing indicators such as pulmonary function, HE staining and Masson staining of lung tissue sections, and the content of hydroxyproline, collagen I, and collagen III in lung tissue. Rats in model group and electroacupuncture group were anesthetized by inhalation of isoflurane at 5% vol and 5 cc/min. After rats were anesthesia, BLM solution diluted in normal saline was instilled through the trachea at a dose of 3 mg/kg. Rats in the normal group were intratracheally instilled with an equal amount of normal saline. This study was reviewed and approved by the Experimental Animal Welfare Ethical Review Committee of Henan University of Traditional Chinese Medicine (IACUC-202406028). The methods of anesthesia and euthanasia for animal experiments adhered to the American Veterinary Medical Association (AVMA) Guidelines for the Euthanasia of Animals (2020).

### Electroacupuncture treatment

On the 15th day after BLM induction, the rats underwent electroacupuncture treatment for 4 weeks. The electroacupuncture treatment was administered three times a week, once every other day. Three groups of acupoints (bilateral Feishu, Shenshu and Zusanli) were selected (Table 1). After being physically restrained, the rats in the electroacupuncture group underwent electroacupuncture at three groups of acupoints for 5 min each, with a stimulation of 1 mA and 1 Hz current during the procedure. Rats in normal group and model group were not treated. After 4 weeks treatment, rats were anesthetized by intraperitoneal injection of 2% pentobarbital sodium at 40 mg/kg. After the rats were anesthetized, pulmonary function was examined using the PFT system. Subsequently, the abdominal cavity of the rats was exposed, and blood was collected from the abdominal aorta. Finally, the thoracic cavity of the rats was exposed, and lung tissue was collected for further analysis. The rat blood was left at room temperature for 4 h, followed by centrifugation at 3000 r/min for 10 min to separate the serum.

**Table 1:** The location and electroacupuncture method of rat acupoint

Acupoint	Number	Location	Method
Feishu	B13	3 mm apart under the spinous process of the third thoracic vertebra on the back	5 mm straight penetration
Shenshu	B23	3 mm apart under the spinous process of the second lumbar vertebra	5 mm straight penetration
Zusanli	S36	Posterolateral side of the knee joint, 5 mm below the capitulum fibulae	5 mm straight penetration

### Body mass

Before administering BLM, the body mass of the rats was measured once and recorded as the body mass on day 0. Subsequently, the body mass of the rats was measured weekly, with all measurements taken at 9:00 AM.

### Pulmonary function test

The lung function testing PFT system was used to test the lung function of the rats. The rat was anesthetized and tracheally intubated. Then, rat was placed in the measurement chamber, and the intubation tube and the respiratory detector were connected. After breathing becomes stable, the vital capacity (VC), lung inspiratory resistance (RI) and dynamic lung compliance (Cydn) of the rat were measured.

### Measuring of TNF-α and IL-4

ELISA was used to measure the levels of TNF-α and IL-4 in rat serum. The specific operation steps were carried out according to the corresponding instructions of the rat IL-4 and TNF-α ELISA kit.

### Measuring of hydroxyproline (HYP)

The alkaline hydrolysis method was used to detect the HYP content. The specific steps were carried out according to the instructions of the HYP Assay Kit.

### Lung tissue pathology

The left lung of the rat was fixed in 4% paraformaldehyde, and embedded in paraffin and sectioned. Lung tissue sections were stained with hematoxylin and eosin. The lung tissue was observed under a light microscope, and the degree of lung tissue inflammation was scored according to Szapiel [23].

Lung tissue sections were stained with aniline blue, and differentiated with 0.2% acetic acid for Masson. The collagen deposition in lung tissue was observed under light microscope, and the degree of pulmonary fibrosis was scored according to Ashcroft [24].

### Immumohistochemical staining

Lung tissue sections were deparaffinized using xylene and alcohol gradients. Citric acid repaired antigen to re-expose the antigen, and 3% H_2_O_2_ blocked endogenous peroxidase activity. BSA blocked non-specific proteins, and COL-I antibody (1:1000) and α-SMA antibody (1:500) were added overnight in the refrigerator at 4 °C. HRP-labeled secondary antibody was added. Hematoxylin counterstained cell nuclei. Image-Pro Plus 6.0 software was used for semi-quantitative analysis, and the integrated optical density (IOD) value was used to reflect the corresponding protein expression content.

### Lung coefficient

After the rats were sacrificed, the whole lungs were removed and the lung wet weight was measured. The formula for calculating lung coefficient was as follows: Lung coefficient (%) = lung wet weight (g)/body mass (kg) × 100%.

### Transcriptomic analysis

Six samples were selected from each group of rats for transcriptomic analysis. The total RNA of rat lung tissue was extracted using the TRIzol method. RNA integrity and total amounts were evaluated using the RNA Nano 6000 Assay Kit on the Bioanalyzer 2100 system. Preparation of the cDNA library, transcriptome sequencing, and preprocessing of data were conducted by Novogene Technology Co., Ltd. with an Illumina NovaSeq 6000 System. Differential expression analysis between two comparison groups was conducted using DESeq2. Criteria for significance were *P*-values < 0.05.

### Proteomic analysis

Six samples were selected from each group of rats for proteomic analysis. 20 mg lung tissue was homogenized in RIPA lysis buffer that contained 1% protease inhibitor cocktail, and then the protein concentration was determined with BCA protein quantification kit. To 200 μg proteins, 4 times volume of ice-cold acetone was added and incubated overnight at − 20 °C. Then the precipitated proteins were dissolved with 8 M urea, and then 20 mM dithiothreitol and 40 mM iodoacetamide were successively added for reduction and alkylation. After diluting with 50 mM NH_4_HCO_3_, the proteins were digested with trypsin overnight. Finally, the peptides were desalted with C18 cartridge column.

The digested peptides were analyzed by Ultimate 3000 NanoLC Q-Exactive mass spectrometer (Thermo Fisher Scientific). A self-packed capillary C18 column (75 μm * 20 cm, 3 μm, 30 nm) was used for separation. The mobile phase A and B were 0.1% FA/H_2_O and 90% ACN/0.1% FA, and the flow rate was 300 nL/min. The elution condition was as follows: 0–3.5 min, 3–7% B; 3.5–45 min, 7–25% B; 45–55 min, 25–35% B; 55–57 min, 35–100% B; 57–59 min, 100% B; 59–61 min, 100–3%B; 61–71 min, 3% B. The data was acquired in Full MS ddMS2 mode, and the parameters were set as follows: spray voltage: 2.7 kV, scan range of MS: 350–1800 m/z, resolution of MS and MS2: 70,000 and 17,500, automatic gain control target of MS and MS2: 1e6 and 1e5, top N: 15, isolation window: 1.6 m/z, normalized collision energy: 27, excluded charge state: 1, 7, 8, > 8.

The raw data was searched by Proteome Discoverer 2.5 (Thermo Fisher Scientific) against the database of mus musculus (17,146 sequences) downloaded from UniProt (https://www.uniprot.org/). Trypsin was specified as the enzyme with up to two missed cleavage sites allowed. The mass tolerances of precursors and fragment ions were set to 10 ppm and 0.02 Da respectively. Carbamidomethylation of cysteine was defined as fixed modification, and oxidation of methionine was defined as dynamic modification. The false discovery rates (FDR) of peptide-spectrum-match, peptide and protein were all set as less than 1%. The protein ratio between samples was calculated based on the pairwise ratio of peptides assigned to the protein. Differential proteins were determined according to the criterion of *P* < 0.05.

### Functional enrichment analysis

The DAVID database (https://david.ncifcrf.gov/home.jsp) [25] was used for Kyoto Encyclopedia of Genes and Genomes (KEGG) enrichment analysis. The species genus was set to “Rattus norvegicus” and the identifier was set to “official gene symbol”.

### Weighted correlation network analysis (WGCNA) and correlation analysis

Sangerbox 3.0 public platform (http://sangerbox.com/home.html) [26] was used for WGCNA. The network type was set to “unsigned”. Genetic screening was performed with mean standard deviation > 50% and outlier samples were filtered. The OmicShare public platform (https://www.omicshare.com/) was used for correlation analysis.

### Gene set enrichment analysis (GSEA)

GSEA ranked genes by differential expression and tested predefined Gene Sets for enrichment. The local version of GSEA analysis tool (http://www.broadinstitute.org/gsea/index.jsp) was used. The enriched pathways with *P* < 0.05 were chosen for further analysis.

### Statistical analysis

SPSS 25.0 was used for data analysis. The results were expressed as mean ± SD. For data conforming to a normal distribution, one-way analysis of variance was employed. If the variances were equal, the Least Significant Difference was used. If the variances were uneven, the Dunnett’s T3 method was used. For data not conforming to a normal distribution, the Kruskal–Wallis rank sum test was used. For body mass data, statistical analysis was performed using the repeated measures method. Statistical significance was set at *P* < 0.05.

## Results

### Analysis of body mass, lung coefficient and pulmonary function

**Fig. 1:**
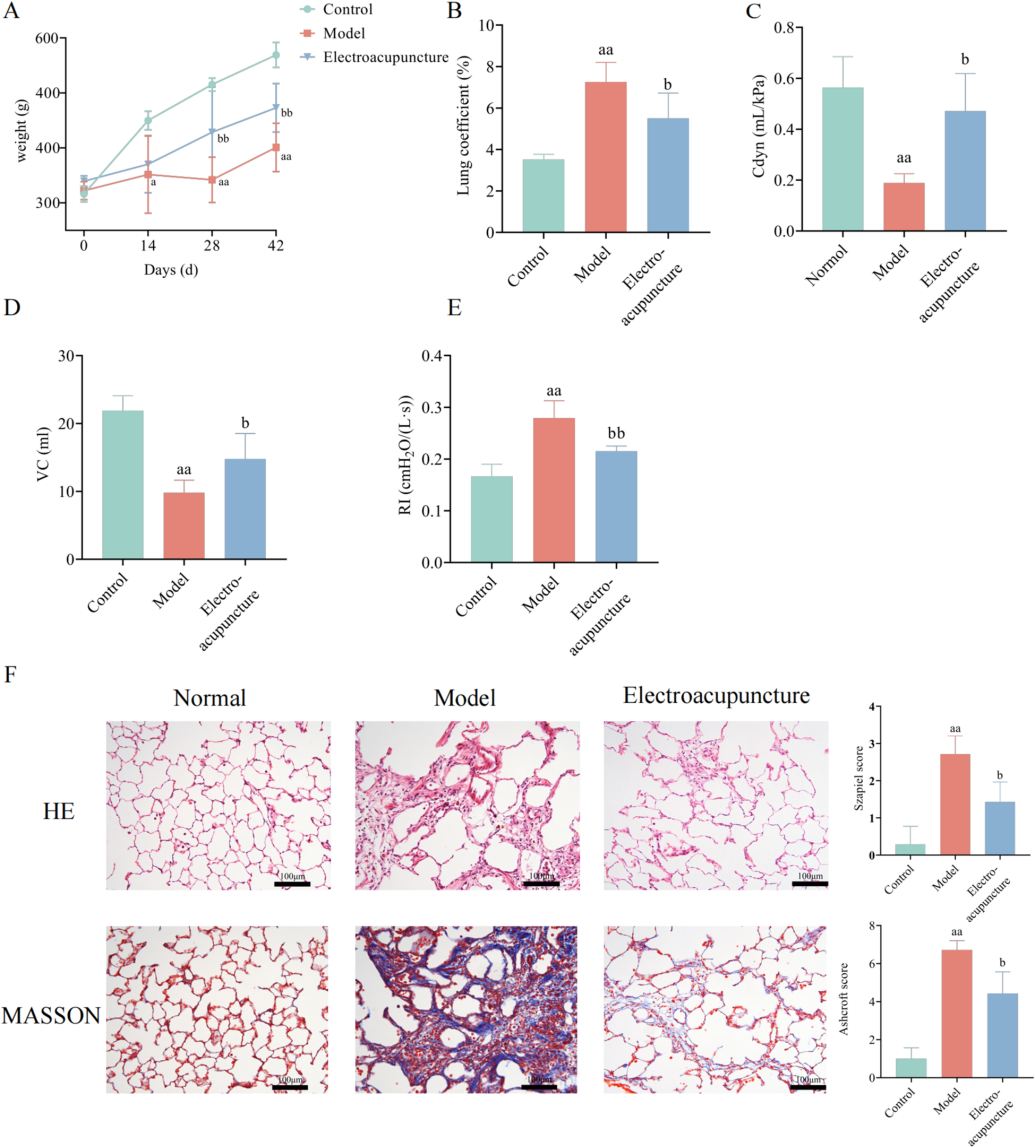
Electroacupuncture improved lung coefficient, pulmonary function and lung tissue injury. **A** changes of body mass in rats. **B** lung coefficient of rats. **C**–**E** Cydn, VC and RI of rats. **F** HE staining (× 200) and Masson staining (× 200) of rats and quantitative analysis. *n* = 6–8. versus Control, a, *P* < 0.05, aa, *P* < 0.01; versus Model, b, *P* < 0.05, bb, *P* < 0.01

The body mass of model group rats and electroacupuncture group rats decreased significantly compared with the rats in the normal group after induced by BLM. Electroacupuncture treatment could improve BLM-induced weight loss in rats (Fig. 1A). The lung coefficient of model group rats was significantly increased compared with normal group rats, and electroacupuncture could reverse the increase in lung coefficient caused by BLM (Fig. 1B). After induced by BLM, compared with normal group rats, the VC and Cdyn of model group rats were reduced, and the RI were increased. Electroacupuncture partially restored the decrease in VC and Cdyn and the increase in RI in rats (Fig. 1C, E).

### Electroacupuncture improved BLM induced lung tissue injury

Lung tissue sections were stained with HE to observe the lung structure. Results showed that BLM destroyed the normal structure of alveoli (Fig. 1F). The alveoli of rats ruptured and fused to form large cavities. The alveolar walls were thickened and a large number of inflammatory cells infiltrated among the alveoli. Electroacupuncture improved the rupture and fusion of alveoli in rats and significantly reduced the infiltration of inflammatory cells. The szapiel score of model group rats increased significantly, which was reversed by electroacupuncture. Masson was used to analyze fibrillar collagen deposition in lung tissue. Collagen deposition was observed in the lung tissue of the model group rats, and electroacupuncture reduced the collagen content in the lung tissue of rats. The ashcroft score of model group rats increased significantly, and decreased in electroacupuncture group rats.

### Electroacupuncture could reduce levels of inflammatory factors in PF rats

**Fig. 2:**
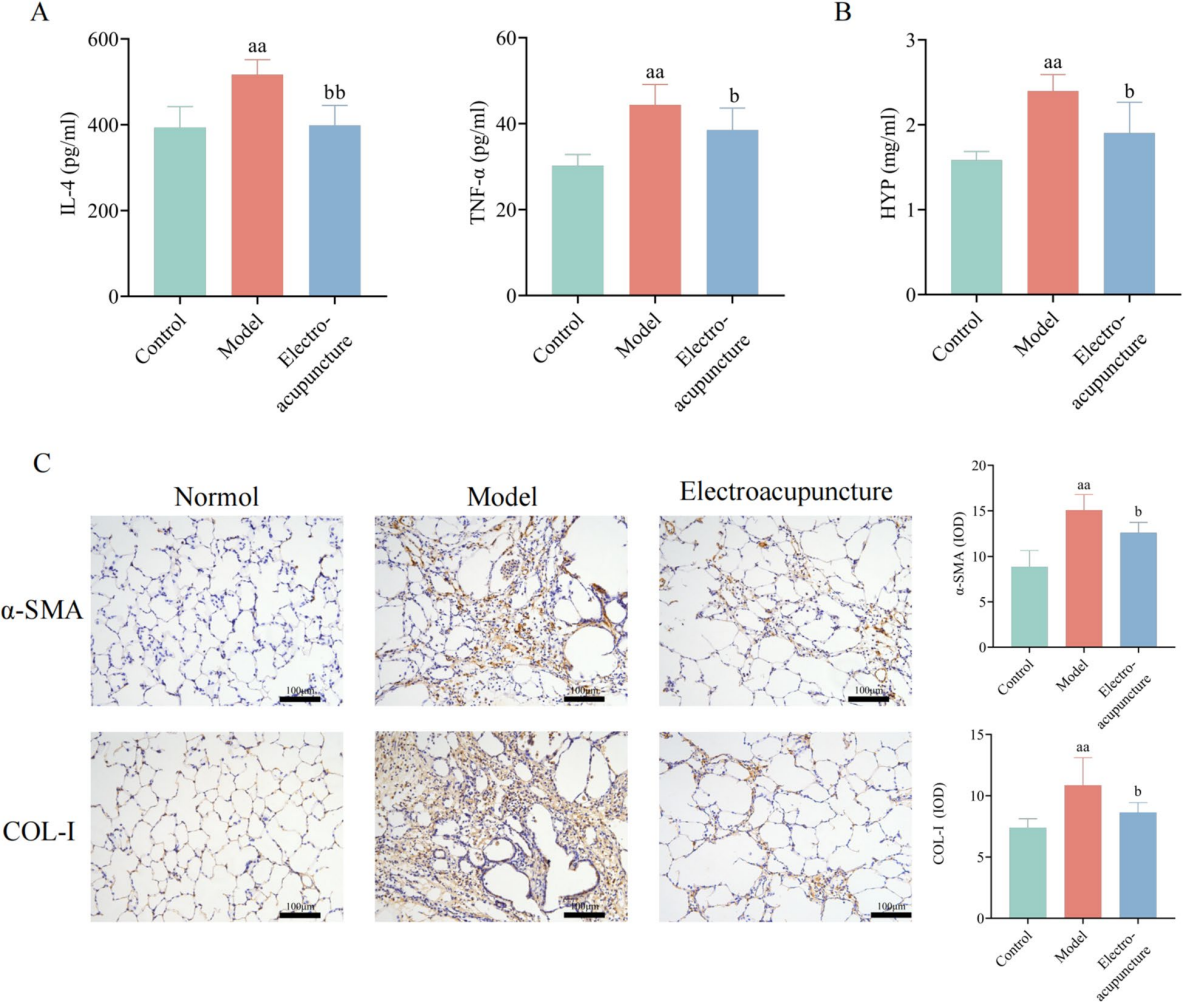
Electroacupuncture reduces inflammatory factors and collagen deposition. **A** IL-4 and TNF-α level of rat serum. **B** HYP level of rat lungs. **C** Col-I (× 200) and α-SMA (× 200) level of rat lungs and quantitative analysis. *n* = 6–8. versus Control, a, *P* < 0.05, aa, *P* < 0.01; versus Model, b, *P* < 0.05, bb, *P* < 0.01

IL-4 and TNF-α in rat serum were used to evaluate the effect of electroacupuncture on inflammation. The results showed that the levels of IL-4 and TNF-α in rat serum increased significantly after induced by BLM. Electroacupuncture could reduce the levels of IL-4 and TNF-α in rat serum (Fig. 2A).

### Electroacupuncture could reduce collagen deposition in PF rats

Next, we detected the levels of HYP, α-SMA, and COL-1 in rat lung tissue to evaluate collagen content. The level of hydroxyproline in the lung tissue of model group rats was significantly increased, and electroacupuncture could improve the increase of HYP caused by BLM (Fig. 2B). Immunohistochemistry showed that extensive positive expression areas were observed in the lung tissue of model group rats, and electroacupuncture could reduce the levels of α-SMA and COL-1 (Fig. 2C). Immunohistochemical quantification results also confirmed these results.

### Differential expression and functional enrichment analysis of transcriptomic

**Fig. 3:**
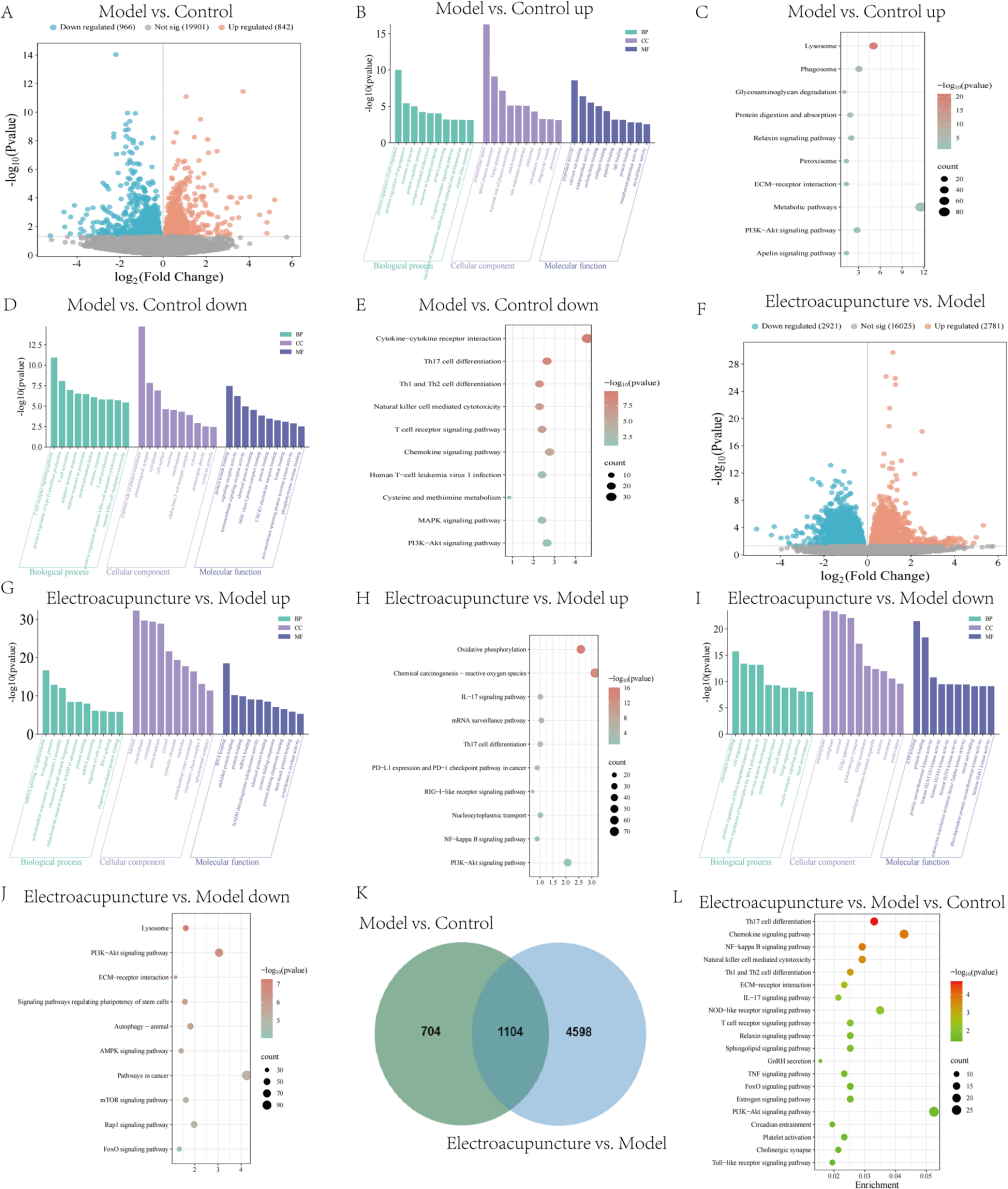
Differential expression and functional enrichment analysis of transcriptome. **A** Volcano plots showing DEGs of Model group and Control group. **B**–**E** GO and KEGG enrichment analysis of downregulated/upregulated DEGs for Model group and Control group. **F** Volcano plots showing DEGs of Electroacupuncture group and Model group. **G**–**J** GO and KEGG enrichment analysis of downregulated/upregulated differential genes for Electroacupuncture group and Model group. **K** Venn diagram of DEGs for Model group, Control group, and Electroacupuncture group. **L** KEGG enrichment analysis of intersecting DEGs for Model group, Control group, and Electroacupuncture group. *n* = 6

In the Model versus Control comparison, 842 upregulated and 966 downregulated differential expressed genes (DEGs) were identified (Fig. 3A). The upregulated genes were enriched in GO terms such as elastic fiber assembly, extracellular space, peroxisome, and collagen binding (Fig. 3B). The KEGG enrichment included pathways like peroxisome, ECM-receptor interaction and PI3K-AKT signaling pathway (Fig. 3C). The downregulated genes were enriched in GO terms such as adaptive immune response, T cell activation, and CXCR3 chemokine receptor binding (Fig. 3D). The KEGG enrichment included pathways like chemokine signaling pathway, MAPK signaling pathway, T cell receptor signaling pathway and PI3K-AKT signaling pathway (Fig. 3E). In the Electroacupuncture versus Model comparison, 2781 upregulated and 2921 downregulated DEGs were identified (Fig. 3F). The upregulated genes were enriched in GO terms such as mitochondrial electron transport, mitochondrial respiratory chain complex I assembly and cytochrome-c oxidase activity (Fig. 3G). The KEGG enrichment included pathways like chemical carcinogenesis-reactive oxygen species, IL-17 signaling pathway, NF-κB signaling pathway and PI3K-AKT signaling pathway (Fig. 3H). The downregulated genes were enriched in GO terms such as cell migration, nervous system development, ATP binding and metal ion binding (Fig. 3I). The KEGG enrichment included pathways like PI3K-AKT signaling pathway, FoxO signaling pathway, AMPK signaling pathway, and ECM-receptor interaction (Fig. 3J). The venn diagram identified 1104 overlapping differentially expressed genes between Model versus Control and Electroacupuncture versus Model (Fig. 3K). Further KEGG enrichment analysis revealed key pathways including PI3K-AKT signaling pathway, FoxO signaling pathway, ECM-receptor interaction and NF-κB signaling pathway (Fig. 3L).

### WCGNA analysis of transcriptomic

**Fig. 4:**
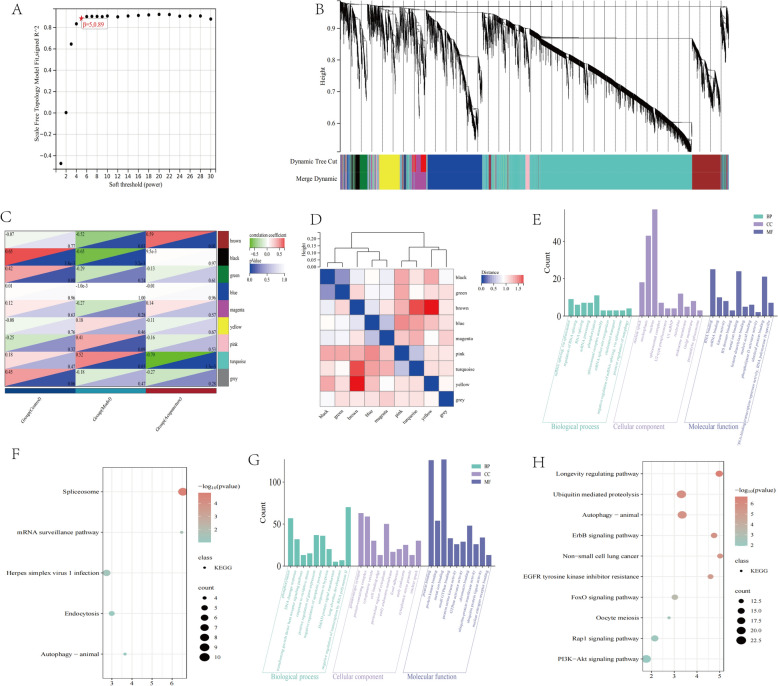
WCGNA analysis of transcriptome. **A** Scale-free network. **B** Gene dendrogram. **C** Module-trait relationships plot of Control group, Model group and Electroacupuncture group. **D** Correlation heat map showing the correlation between the 9 modules. **E**, **F** GO and KEGG enrichment analysis of the brown module. **G**, **H** GO and KEGG enrichment analysis of the turquoise module

In order to further clarify the gene sets that change cooperatively, WGCNA was used to identify relevant modules involved in electroacupuncture intervention in pulmonary fibrosis. When the scale-free fitting index was close to 0.9, the minimum soft threshold suitable for constructing a scale-free network was 5, so 5 was selected as the optimal soft threshold for subsequent analysis (Fig. 4A). A dendrogram of genes with different similarities was generated based on topological overlap and module colors for clustering (Fig. 4B). A total of 9 modules were obtained, and the brown module and turquoise module showed significant correlation with the Model group and Electroacupuncture group (Fig. 4C). Then, there was a clear correlation between the the brown module and turquoise module (Fig. 4D). The genes of brown module were enriched in GO terms such as metal ion binding, positive regulation of autophagy and regulation of RNA splicing (Fig. 4E). The KEGG enrichment analysis for brown module included pathways like mRNA surveillance pathway, endocytosis and autophagy (Fig. 4F). The genes of turquoise module were enriched in GO terms such as response to oxidative stress, metal ion binding, TGF-β receptor signaling pathway (Fig. 4G). The KEGG enrichment analysis for turquoise module included pathways like PI3K-AKT signaling pathway, FoxO signaling pathway, Rap1 signaling pathway and autophagy (Fig. 4H).

### GSEA analysis of transcriptomic

**Fig. 5:**
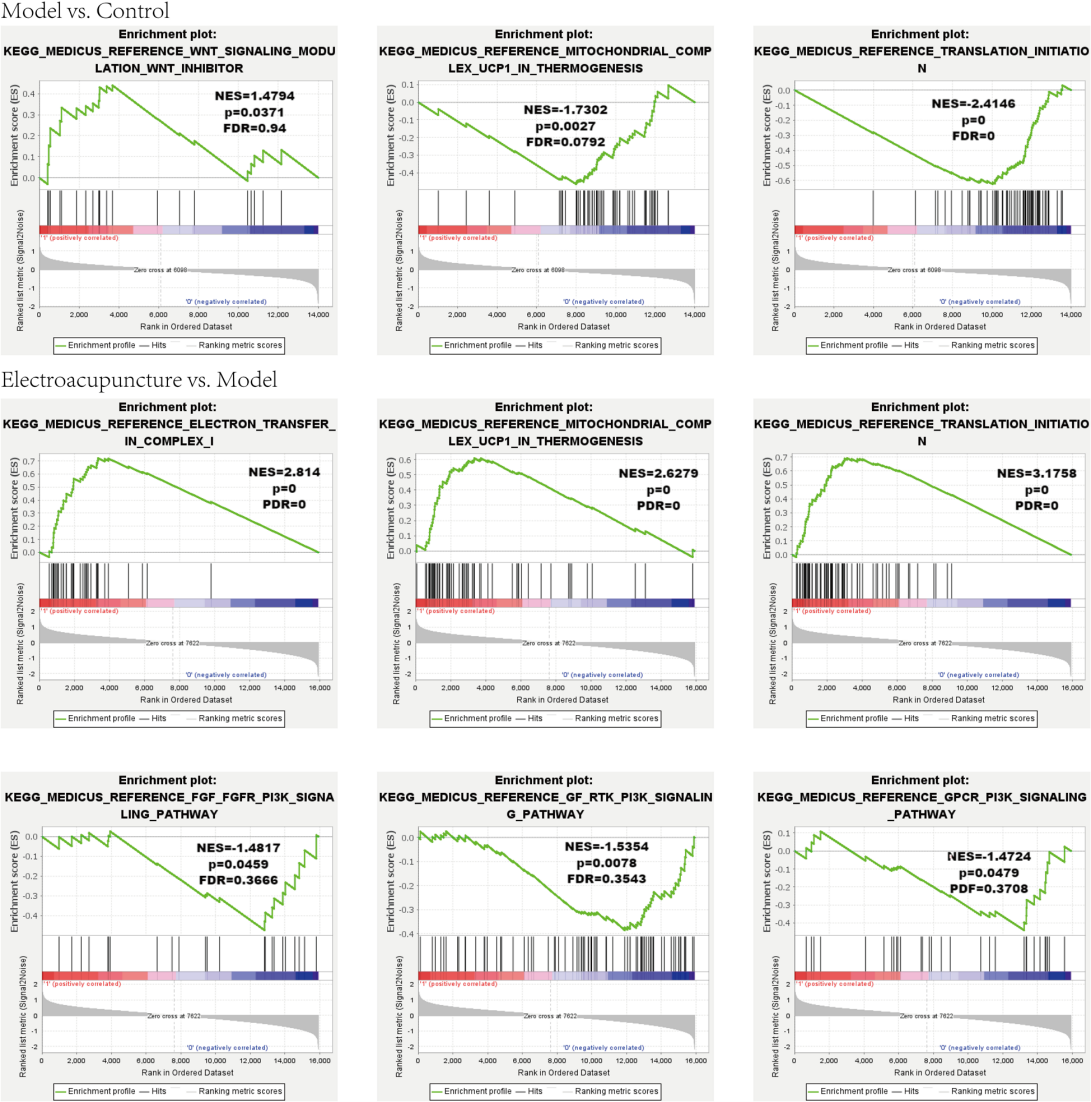
GSEA based on transcriptomics of Control group, Model group and Electroacupuncture group

The enrichment results of GSEA revealed significant regulation in Wnt signaling modulation Wnt inhibitor (NES = 1.4794, *P* = 0, FDR = 0.94), mitochondrial complex UCP1 in thermogenesis (NES = − 1.7302, *P* = 0.0027, FDR = 0.0792) and translation initiation (NES = − 2.4146, *P* = 0, FDR = 0) in Model group versus Control group (Fig. 5). Then, in Electroacupuncture group versus Model group, significant regulation was identified in electron transfer in complex I (NES = 2.814, *P* = 0, FDR = 0), mitochondrial complex UCP1 in thermogenesis (NES = 2.6279, *P* = 0, FDR = 0), translation intiation (NES = 3.1758, *P* = 0, FDR = 0), FGF-FGFR-PI3K signaling pathway (NES = − 1.4817, *P* = 0.0459, FDR = 0.3666), GF-RTK-PI3K signaling pathway (NES = − 1.5354, *P* = 0.0078, FDR = 0.3543) and GPCR-PI3K signaling pathway (NES = − 1.4724, *P* = 0.0479, FDR = 0.3708) (Fig. 5).

### Differential expression and functional enrichment analysis of proteomics

**Fig. 6:**
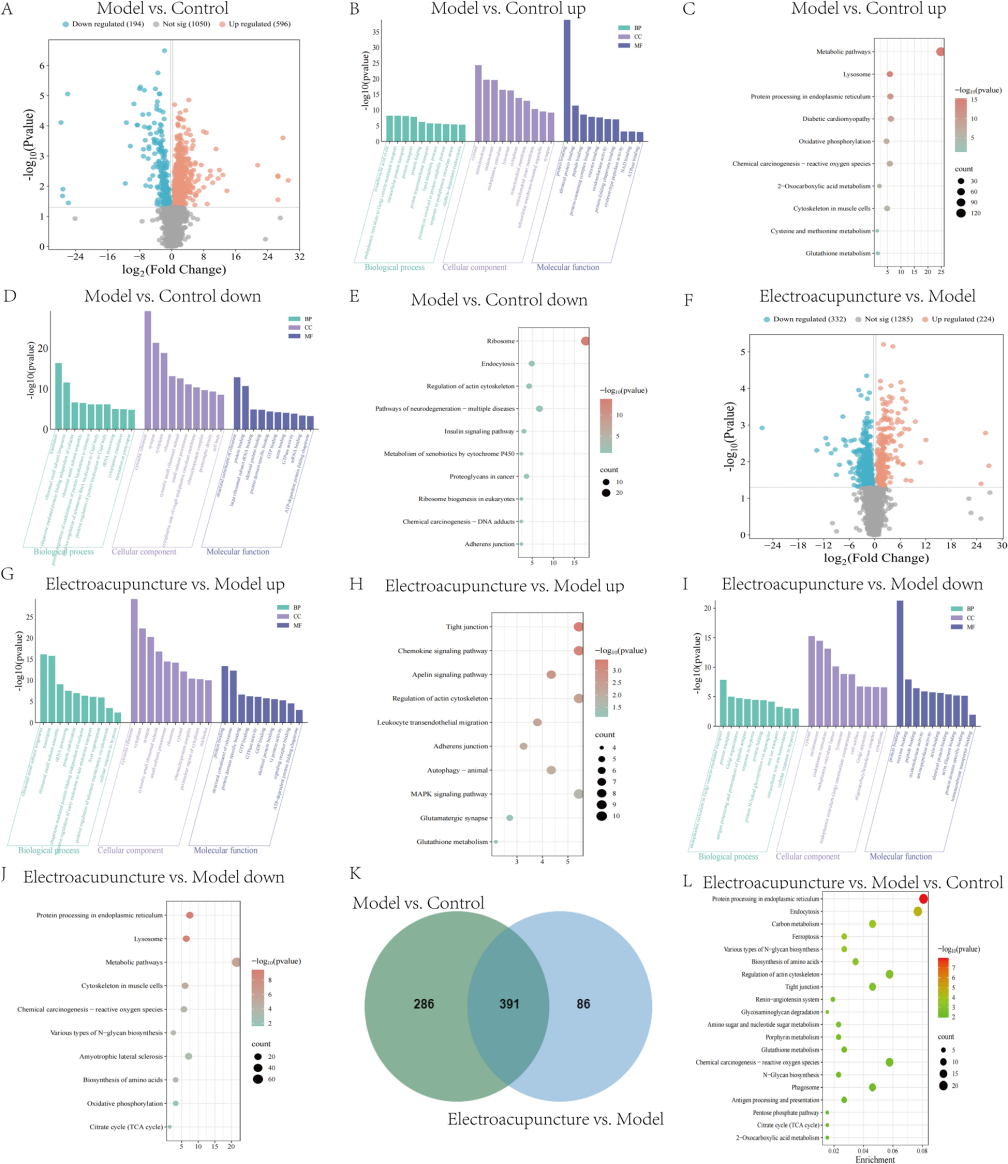
Differential expression and functional enrichment analysis of proteomics. **A** Volcano plots showing DEPs of Model group and Control group. **B**–**E** GO and KEGG enrichment analysis of downregulated/upregulated DEPs for Model group and Control group. **F** Volcano plots showing DEPs of Electroacupuncture group and Model group. **G**–**J** GO and KEGG enrichment analysis of downregulated/upregulated DEPs for Electroacupuncture group and Model group. **K** Venn diagram of DEPs for Model group, Control group, and Electroacupuncture group. **L** KEGG enrichment analysis of intersecting DEPs for Model group, Control group, and Electroacupuncture group. *n* = 6

In the Model versus Control comparison, 596 upregulated and 194 downregulated differential expression proteins (DEPs) were identified (Fig. 6A). The upregulated proteins were enriched in GO terms such as oxidoreductase activity, lipid metabolic process, and clathrin-dependent endocytosis collagen binding (Fig. 6B). The KEGG enrichment included pathways like metabolic pathway, chemical carcinogenesis-reactive oxygen species and glutathione metabolism (Fig. 6C). The downregulated proteins were enriched in GO terms such as translation, ATP-dependent protein folding chaperone (Fig. 6D). The KEGG enrichment included pathways like endocytosis, insulin signaling pathway and adherens junction (Fig. 6E). In the Electroacupuncture versus Model comparison, 224 upregulated and 332 downregulated DEPs were identified (Fig. 6F). The upregulated proteins were enriched in GO terms such as translation, cellular response to hypoxia and ATP-dependent protein folding chaperone (Fig. 6G). The KEGG enrichment included pathways like glutathione metabolism, chemokine signaling pathway, MAPK signaling pathway and autophagy (Fig. 6H). The downregulated proteins were enriched in GO terms such as iron ion transport, response to hypoxia, and oxidoreductase activity (Fig. 6I). The KEGG enrichment included pathways like chemical carcinogenesis-reactive oxygen species, metabolic pathways (Fig. 6J). The venn diagram identified 391 overlapping DEPs between Model versus Control and Electroacupuncture versus Model (Fig. 6K). Further KEGG enrichment analysis revealed key pathways including ferroptosis, glutathione metabolism, endocytosis, and chemical carcinogenesis-reactive oxygen species (Fig. 6L).

### Multi-omics joint analysis of transcriptomic and proteomic

**Fig. 7:**
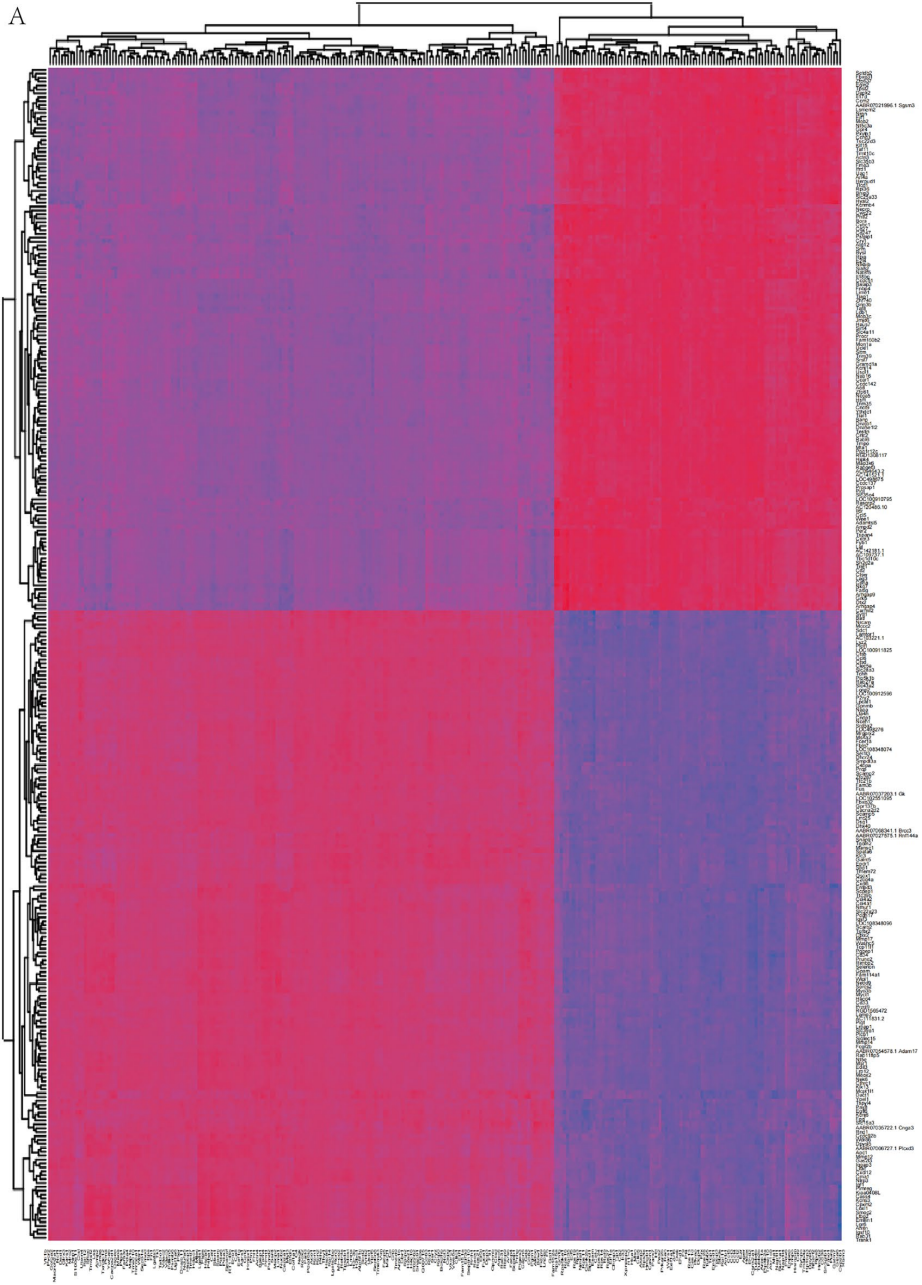

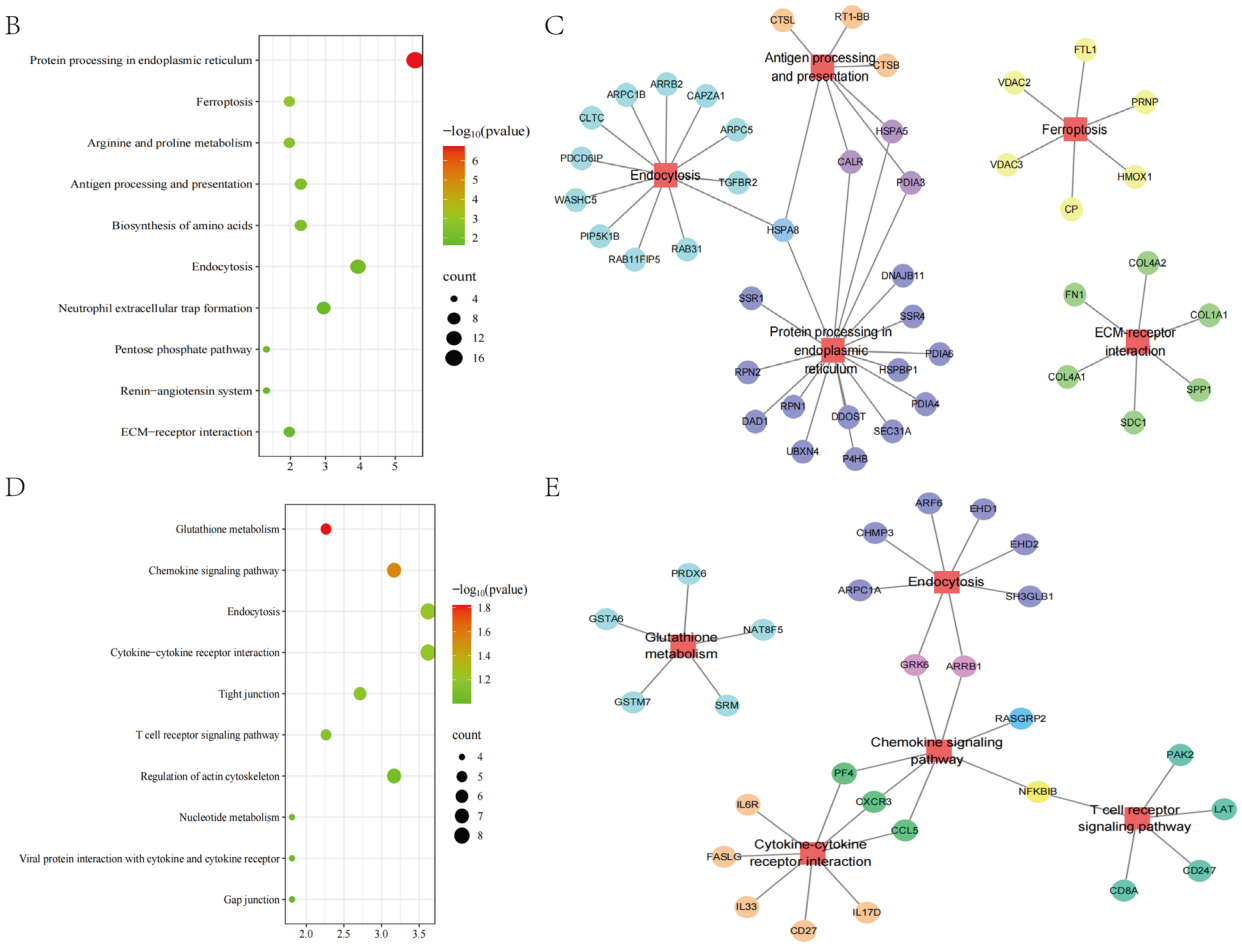
**A** Heap map showing association between identified differential genes and proteins. **B**, **C** KEGG enrichment analysis and molecular networks of genes and proteins up-regulated in the Model group and down-regulated in the Electroacupuncture group. **D**, **E** KEGG enrichment analysis and molecular networks of genes and proteins down-regulated in the Model group and up-regulated in the Electroacupuncture group

Correlation analysis was performed using Pearson correlation coefficient to assess the degree of association between the identified differential genes and proteins, and (Fig. 7A). 300 differential genes and 265 differential proteins were identified a strong correlation. KEGG enrichment analysis of genes and proteins up-regulated in the Model group and down-regulated in the Electroacupuncture group showed that ferroptosis, endocytosis, ECM-receptor interaction, protein processing in endoplasmic reticulum and antigen processing and presentation were significantly enriched (Fig. 7B). Molecular networks reveal the regulatory genes and proteins of these signaling pathways (Fig. 7C). Then, KEGG enrichment analysis of genes and proteins down-regulated in the Model group and up-regulated in the Electroacupuncture group showed that glutathione metabolism, chemokine signaling pathway, cytokine-cytokine receptor interaction, T cell receptor signaling pathway and endocytosis were significantly enriched (Fig. 7D). Molecular networks also reveal the regulatory genes and proteins of these signaling pathways (Fig. 7E).

### Electroacupuncture improved iron overload and lipid peroxidation in pulmonary fibrosis rat

**Fig. 8:**
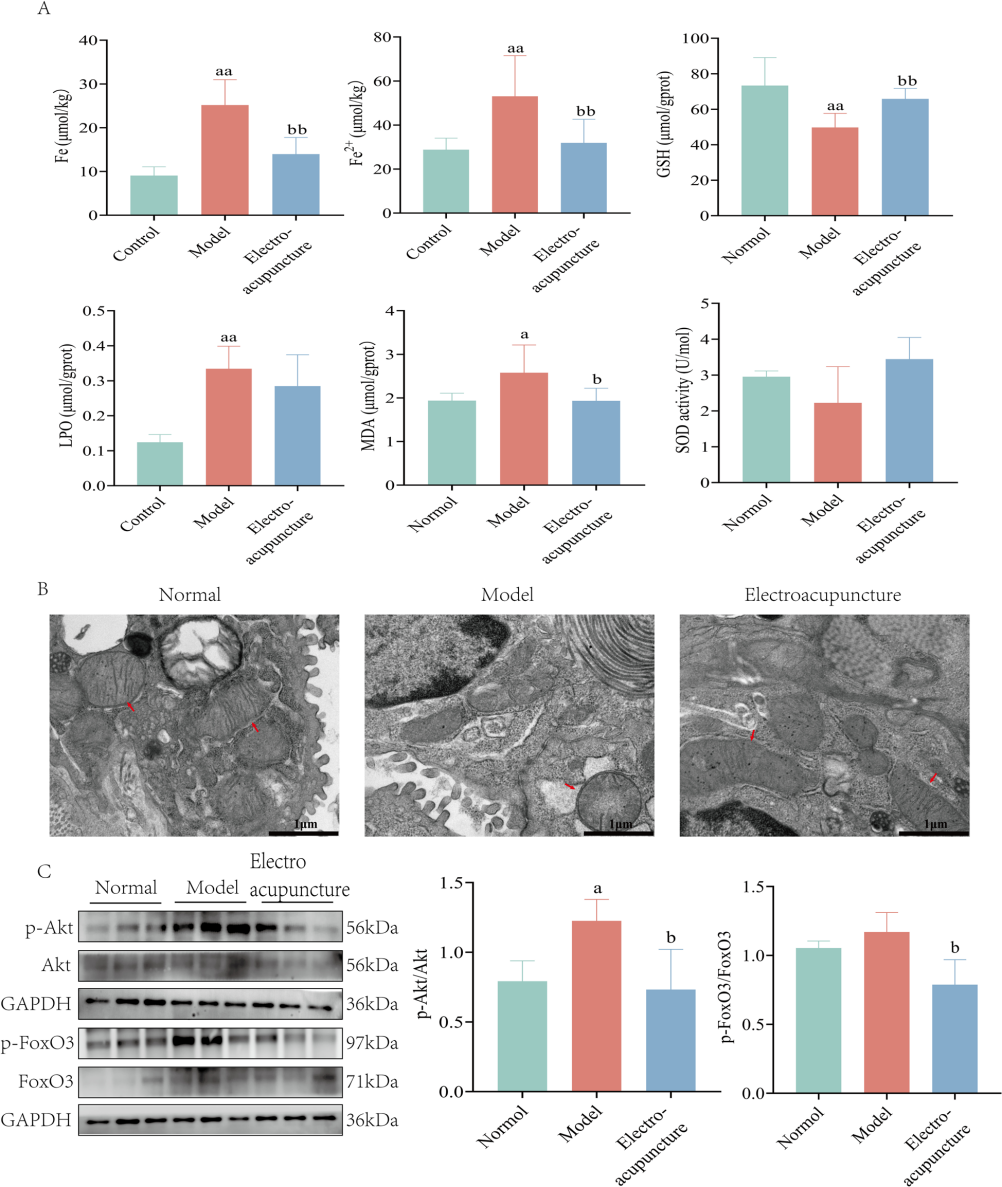
**A** The contents of Fe^3+^, Fe^2+^, GSH, LPO, MDA and SOD in the lung tissue of pulmonary fibrosis rats. **B** Ultrastructural changes of mitochondria (red arrow) in lung tissue of pulmonary fibrosis rats (× 30,000). **C** Levels of p-Akt and p-FoxO3. *n* = 6–8. versus Control, a, *P* < 0.05, aa, *P* < 0.01; versus Model, b, *P* < 0.05, bb, *P* < 0.01

To elucidate the mechanism of electroacupuncture in pulmonary fibrosis, iron overload and lipid peroxidation were evaluated. The results showed that, in pulmonary fibrosis rats, the contents of Fe^3+^, Fe^2+^, Lipid Peroxide (LPO) and malondialdehyde (MDA) were significantly increased, and the content of glutathione (GSH)was significantly reduced. In addition, the content of superoxide dismutase (SOD) was also reduced, although there was no statistical difference. On the contrary, electroacupuncture intervention reduced the contents of Fe^3+^, Fe^2+^, LPO and MDA, and increased the contents of GSH and SOD in the lung tissue of pulmonary fibrosis rats (Fig. 8A).

The mitochondria of the rats in the control group were moderate in size, with complete structure, and the inner mitochondrial cristae structure was clear and attached with grana. Then, in the model group, the mitochondria became smaller, and the outer membrane was broken. The number of inner cristae and grana was also reduced. After the electroacupuncture treatment, the mitochondria had normal shape and inner mitochondria. The cristae structure was complete and the number of grana was increased (Fig. 8B). WB demonstrated that the level of p-Akt in the lung tissues of rats in the model group was significantly elevated, while electroacupuncture treatment inhibited the level of p-Akt. Similarly, electroacupuncture treatment also suppressed the level of p-FoxO3 (Fig. 8C).

## Discussion

This study is dedicated to elucidating the characteristics of electroacupuncture in improving pulmonary fibrosis. We combine transcriptomics and proteomics to confirm the role of electroacupuncture in regulating collagen deposition and enhancing pulmonary function for the first time. These effects are related to ferroptosis, PI3K-AKT signaling pathway.

Currently, electroacupuncture treatment has received increasing attention for its role in pulmonary diseases due to its safety and effectiveness. A systematic review has shown that electroacupuncture has positive effects on lung function, exacerbation status, 6-minute walking distance, mMRC score, and CAT score in patients with COPD [27]. Wu J et al. find electroacupuncture alleviates cytokine storm‑secondary lung injury in CpG1826‑challenged mice by regulating the P2X7‑dependent CD39‑NLRP3 pathway. First, in this study, we confirm that electroacupuncture can improve the deterioration of VC, RI, and Cydn in rats caused by BLM [28]. Decline in VC is the main indicator of the progression of pulmonary fibrosis. 70% of pulmonary fibrosis patients experience a decrease in VC to varying degrees within 3 years [29]. The latest research shows that VC is used as a surrogate indicator of mortality in pulmonary fibrosis patients [30]. There is a strong positive correlation between the decrease of VC and 52-week mortality in pulmonary fibrosis patients. Decreased lung tissue elasticity and pulmonary resistance, especially decreased dynamic lung compliance and increased RI, are important factors in dyspnea in pulmonary fibrosis [31]. Fu et al. successfully induce a mouse pulmonary fibrosis model with 1-nitropyrene and confirmed that RI in mice significantly increases and Cydn decreases after 1-nitropyrene treatment [32]. Consistent with these studies, our study confirms that electroacupuncture can improve these indicators to alleviate BLM-induced decrease in respiratory capacity.

Pulmonary fibrosis is a disease driven by a combination of alveolar collapse and collagen deposition [33]. Excessive collagen deposition is a remarkable feature of pulmonary fibrosis. Col I levels are significantly increased in patients with pulmonary fibrosis, and obvious changes can occur in the early stages of the disease [34]. Recent studies have shown that the dynamic remodeling process of collagen, namely degradation and renewal, is also widely present in patients with pulmonary fibrosis, which promotes the progression of pulmonary fibrosis [35]. In our study, we also find that electroacupuncture can improve the alveolar destruction caused by BLM and reduce the accumulation of collagen in the lung tissue. HYP, as a unique amino acid to collagen and one of the main components of collagen, is often used to evaluate the degree of collagen deposition [36]. In our study, consistent with previous studies, electroacupuncture significantly reverses the BLM-induced HYP levels in rat lung tissue. The differentiation of lung fibroblasts into myofibroblasts is a key step in pulmonary fibrosis [37]. During this process, a large amount of collagen is secreted into the lung tissue, resulting in excessive collagen deposition. α-SMA is one of the markers of myofibroblasts [38]. We find that electroacupuncture reduces the expression of α-SMA in rat lung tissue, suggesting that electroacupuncture may prevent the differentiation process of fibroblasts.

Ferroptosis is a widely concerned form for regulating necrotic cell death that has been reported to play an important role in a variety of diseases, including cancer, neurodegeneration, and ischemic organ damage [39]. Increasing evidence suggests its potential function in pulmonary fibrosis. Ferroptosis inhibitor liproxstatin-1 and the iron chelator deferoxamine alleviates the symptoms of pulmonary fibrosis induced by BLM, suggesting the underlying role for ferroptosis in pulmonary fibrosis [40]. Ferroptosis refers to iron-dependent cell death caused by excess lipid peroxidation leading to plasma membrane rupture [41]. When ferroptosis occurs, both iron accumulation and lipid peroxidation can lead to oxidative damage to the plasma membrane [42]. Increased iron accumulation is a key trigger of ferroptosis, and transferrin mediates iron uptake through the transferrin receptor [43]. Moreover, intracellular iron storage protein and iron export transporter solute carrier family 40 member 1 are degraded through autophagy, increasing iron accumulation, thereby enhancing ferroptosis [44]. Excess intracellular iron can promote subsequent lipid peroxidation through the generation of reactive oxygen species and activation of iron-containing enzymes such as arachidonic acid lipoxygenases [45]. Ferroptosis is mainly a balance process between oxidative damage and antioxidant [46]. The GSH-glutathione peroxidase 4 (GPX4) antioxidant system plays an important role in protecting cells from ferroptosis. The xc system is responsible for importing cyst(e)ine as the rate-limiting substrate for GSH synthesis to exchange intracellular glutamate [47]. GPX4 uses GSH as a reducing cofactor to reduce PLOOH to fatty alcohols, thereby inhibiting cellular ferroptosis [48]. In previous studies, empagliflozin is found to modulate the sestrin2/AMPK/Nrf2/HO-1 signaling pathway, targeting ferroptosis and autophagy to counteract BLM-induced PF [49]. In our study, we also find that electroacupuncture can significantly reduce the content of Fe^3+^ and Fe^2+^ in the lung tissue of pulmonary fibrosis rats, thereby improving iron overload. Moreover, electroacupuncture reduces the contents of LPO and MDA, increases the contents of GSH and SOD, thereby balancing oxidative and antioxidant damage, improving lipid peroxidation and inhibiting ferroptosis. However, it must be acknowledged that our current findings can only demonstrate the intervention effect of electroacupuncture on ferroptosis and pulmonary fibrosis. Whether electroacupuncture intervenes in pulmonary fibrosis through ferroptosis requires further investigation to verify, such as the application of ferroptosis inhibitors.

PI3K/Akt signaling is involved in various biological processes such as cell proliferation, apoptosis inhibition, cell migration, and vesicle transport [50]. Previous studies have shown that regulating PI3K/Akt signaling can enhance the activation of GPX4/SLC7A11 signaling, thereby inhibiting ferroptosis [51]. Transcriptome and proteome results reveal that PI3K/Akt signaling is significantly activated in the model group, while electroacupuncture inhibites PI3K/Akt signaling, suggesting that PI3K/Akt signaling may be one of the key signals for electroacupuncture to regulate ferroptosis. Furthermore, we found that electroacupuncture can reduce the phosphorylation level of Akt by detecting the levels of Akt and p-Akt proteins. FoxO3 is also involved in the process of ferroptosis. Previous studies have shown that inhibiting FoxO3 activity can reduce the levels of ROS, MDA and Fe^2^⁺, while increasing the levels of GSH and GPX4 in tissues or cells, thereby suppressing the ferroptosis process [52]. In our study, electroacupuncture treatment is also found to suppress FoxO3 levels, thereby inhibiting the ferroptosis process.

This study combines transcriptomics and proteomics to identify the gene profile and protein profile of electroacupuncture intervention in pulmonary fibrosis. The efficacy characteristics of electroacupuncture in treating mice with BLM-induced pulmonary fibrosis were evaluated. Our study provides a basis for electroacupuncture in the treatment of pulmonary fibrosis and may beneficial to the widespread application of electroacupuncture in the treatment of pulmonary fibrosis. Combining electroacupuncture with drug therapy may enable patients to achieve better therapeutic effects. Moreover, benefiting from the minimal side effects of electroacupuncture, patients are more likely to accept this treatment modality. However, it is undeniable that there are still enormous challenges for the widespread clinical application of electroacupuncture in the treatment of pulmonary fibrosis. The standardization of electroacupuncture treatment (including frequency, intensity, treatment duration, and course of treatment) has not yet been established. Additionally, the differences in therapeutic effects of electroacupuncture at different stages of the disease are worthy of further investigation to clarify the indications for electroacupuncture treatment. Furthermore, most existing clinical studies on electroacupuncture are small-sample, single-center trials, and there is a need to conduct multi-center, large-sample randomized controlled trials to evaluate its efficacy.

## Conclusions

In this study, we evaluate the characteristics of electroacupuncture in treating BLM-induced pulmonary fibrosis in mice, and combine transcriptomics and proteomics to identify the gene and protein profiles responsible for the efficacy of electroacupuncture. Ferroptosis and PI3K-AKT signaling pathway are potential signals for electroacupuncture to intervene in pulmonary fibrosis. But what we need to acknowledge and improve is that the mechanism of electroacupuncture needs more molecular biology experiments to verify.

## Data Availability

The datasets used and/or analysed during the current study are available from the corresponding author on reasonable request.

## Abbreviations

IPF: Idiopathic pulmonary fibrosis
COL-I: Collagen I
α-SMA: α-Smooth muscle actin
HYP: Hydroxyproline
BLM: Bleomycin
ECM: Extracellular matrix
LPO: Lipid peroxide
MDA: Malondialdehyde
GSH: Glutathione
SOD: Superoxide dismutase
VC: Vital capacity
RI: Lung inspiratory resistance
Cydn: Dynamic lung compliance
